# Dissection of leucine-rich repeat receptor-like protein kinases: insight into resistance to *Fusarium* wilt in tung tree

**DOI:** 10.7717/peerj.14416

**Published:** 2022-11-18

**Authors:** Yunpeng Cao, Tingting Fan, Bo Zhang, Yanli Li

**Affiliations:** 1 School of Health and Nursing, Wuchang University of Technology, Wuhan, China; 2 School of Forestry, Central South University of Forestry and Technology, Changsha, China; 3 Wuhan Botanical Garden, Chinese Academy of Sciences, Wuhan, China

**Keywords:** LRR-RLK, Tung tree, Expression patterns, Evolution analysis

## Abstract

The tung tree is a woody oil plant native to China and widely distributed in the subtropics. The three main species commonly known as *Vernicia* are *V. fordii*, *V. montana*, and *V. cordata*. The growth and development of *V. fordii* are affected by a large number of plant pathogens, such as *Fusarium* wilt caused by *Fusarium* sp. In contrast, *V. montana* shows significant resistance to *Fusarium* wilt. The leucine-rich repeat receptor-like protein kinase (LRR-RLK) is the largest class of receptor-like kinases associated with plant resistance to *Fusarium* wilt. Here, we identified 239 *VmLRR-RLKs* in *V. montana*, and found that there were characteristic domains of resistance to *Fusarium* wilt in them. Phylogenetic analysis suggested that the *VmLRR-RLKs* are divided into 14 subfamilies, indicating that homologous genes in the same group may have similar functions. Chromosomal localization analysis showed that *VmLRR-RLKs* were unevenly distributed on chromosomes, and segment duplications were the main reason for the expansion of *VmLRR-RLK* family members. The transcriptome data showed that six orthologous pairs were up-regulated in *V. montana* in response to *Fusarium* wilt, while the corresponding orthologous genes showed low or no expression in *V. fordii* in resistance *Fusarium* wilt, further indicating the important role of *LRR-RLKs* in *V. montana*’s resistance to infection by *Fusarium* spp. Our study provides important reference genes for the future use of molecular breeding to improve oil yield and control of *Fusarium* wilt in tung tree.

## Introduction

The tung tree is an important industrial oil tree species in the world. The three most important tung tree species in the world are *Vernicia fordii*, *V. montana* and *V. cordata* (Haw, 2017; Stuppy et al., 1999). Where *V. fordii* is widely planted because of its high oil production (Cui et al., 2018). However, compared with the other two tree species, the growth and development of *V. fordii* are more susceptible to *Fusarium* wilt (Cao et al., 2021; Chen et al., 2016). On the contrary, *V. montana* showed significant resistance to *Fusarium* wilt (Chen et al., 2016; Jiang et al., 2022a; Zhang et al., 2016).

In the process of plant growth and development, cell-environment signals and cell-cell interaction signals can stimulate and induce plants to produce a variety of different signal transduction pathways. The reversible phosphorylation regulation mechanism of protein kinases (PK) is involved in the process of cell signal transduction (Cao et al., 2021). Receptor-like kinase (RLK) family members can sense and process external or internal signals in living cells of plants (Gou et al., 2010; Shiu & Bleecker, 2001). The first plant *leucine-rich repeat receptor-like kinase* (*LRR-RLK*) gene was cloned and found in maize (Walker & Zhang, 1990). The researchers then found that *LRR-RLKs* are widely distributed in plant genomes and have expanded to hundreds of members per genome, such as 309 members in rice, 303 in *Brassica rapa*, and 379 in poplar (Hwang, Kim & Jang, 2011; Rameneni et al., 2015; Zan et al., 2013). The typical plant LRR-RLKs proteins contain three characteristic domains: extracellular domain (ECD), transmembrane domain (TM), and intracellular kinase domain (KD) (Gou et al., 2010; Shiu & Bleecker, 2001; Song et al., 2017).

At present, the functions of *LRR-RLKs* in many plants, especially model plants, have been fully studied (Ariza-Suarez et al., 2022; Gottin et al., 2021). For instance, brassinolide-insensitive 1 (BRI1) plays a key role in a variety of plant growth and development processes by sensing the steroid hormone brassinolide (BRs) (Nolan, Chen & Yin, 2017). PXC1 from *Arabidopsis*, an LRR-RLK protein, is essential to regulate secondary wall formation (Wang et al., 2013). The *Arabidopsis LRR-RLK*, *HSL3*, is a regulator of the drought stress response and stomatal closure correlated with hydrogen peroxide homeostasis (Liu et al., 2020). *COE1*, also known as *LRR-RLK*, plays a critical role in the formation of commissural patterns in rice (Sakaguchi et al., 2010). In addition, studies have confirmed that the RECEPTOR-LIKE PROTEIN KINASE 1 (RPK1) acts as a defense-related receptor in *Oryza rufipogon*, while the *Arabidopsis* homolog of RPK1, AtRPK1, has also reported to play key roles in leaf senescence and drought stress responses (Law et al., 2012; Lee et al., 2011; Osakabe et al., 2005).

Recently, systematic identification of *LRR-RLKs* has been carried out in five Rosaceae species (Sun et al., 2017), two citrus species (Magalhães et al., 2016), *Solanum lycopersicum* (Wei et al., 2015), *Amborella trichopoda* (Liu et al., 2016), and other plant species (Liu et al., 2017; Sun et al., 2018; Wang et al., 2019). However, it is still excluded whether the function of *LRR-RLKs* as alarm genes in tung tree in response to *Fusarium* wilt infection. In our study, we used the *Fusarium* wilt-susceptible *V. fordii* and *Fusarium* wilt-resistant *V. montana* as materials to study the genetic mechanisms of *LRR-RLKs* in resistance to *Fusarium* wilt infection in tung tree. Our data might provide important candidate genes for future molecular-assisted breeding in tung tree.

## Materials and Methods

### Database search

The proteins, CDSs, and GFF files of *V. montana* and *V. fordii* were obtained from NCBI database, as described by Cui et al. (2018), Li et al. (2022) and Cao et al. (2022). The proteins and CDSs of LRR-RLKs in *V. fordii* were obtained from Cao et al. (2021) and Cao et al. (2020). Subsequently, we used InterProScan (Jones et al., 2014) to identify distinct protein signatures in these predicted datasets and developed a local database to analyze the datasets in each included plant genome. Pkinase (PF00069) and Pkinase_Tyr (PF07714) are considered to be KD domains specific to LRR-RLK proteins. LRR domains containing LRRNT_2 (PF08263), LRRCT (PF01463), LRV (PF01816), LRRNT (PF01462), LRR_1 (PF00560), LRR_2 (PF07723), LRR_3 (PF07725), LRR_4 (two copies; PF12799), LRR_5 (six copies; PF13306), LRR_8 (PF13855) and LRR_9 (PF14580) are LRR-RLK-specific R domains. To detect the candidate *LRR-RLKs* in *V. montana*, we first downloaded and obtained their HMM models from the Pfa, website (El-Gebali et al., 2019). The local database was then retrieved using the HMM model using the HMMER 3.0 software (Mistry et al., 2013). Each amino acid sequence of *V. fordii* VfLRR-RLKs was also used as a query to determine LRR-RLKs in the *V. montana* local genome database using BLASTp with an E-value less than 1e−5. Finally, if identified candidate genes contained complete KD and R domains, we considered them to be candidate LRR-RLKs.

### Phylogenetic analysis

All full-length VmLRR-RLK proteins were subjected to multiple sequence alignment using MAFFT (version 7) software with default parameters (Katoh & Standley, 2013). MEGA (version 5) software was used to construct the neighbor-joining tree (Tamura et al., 2011). The bootstrap value is an important method to analyze the reliability of the phylogenetic tree, so to confirm the reliability of the phylogenetic relationship, this study used 1,000 bootstrap values to test the reliability of this tree. The iTOL website and FigTree software were used to edit and visualize the phylogenetic tree (Letunic & Bork, 2019).

### Gene structure and collinear analysis

The information about the starting position of each *VmLRR-RLK* on the chromosome was obtained from the GFF file of the *V. montana* genome, and then TBtools was used to visualize the location of the *VmLRR-RLK* gene on the chromosomes (Chen et al., 2018). The DNA and CDS sequence of each *VmLRR-RLK* gene were obtained from the *V. montana* genome and compared using Tbtools (version 1.098769) software to obtain the gene structure information of *VmLRR-RLK*. The collinear analysis was identified among genome regions by MCScanX software with an E-value of 1e−10 (Wang et al., 2012), as described by Jiang et al. (2022b). The TBtools (version 1.098769) software was carried out to visualize the collinear relationships (Chen et al., 2018).

### Transcriptome analysis

Transcriptome data (PRJNA445068, PRJNA483508, and PRJNA318350) were collected and retrieved from NCBI databases to analyze expression patterns. SRA toolkit was used to decompress raw data into fastq format. Then, each dataset was mapped into the corresponding reference genome using HISAT2 using default parameters (Kim et al., 2019; Pertea et al., 2016). The expression levels of genes were calculated using StringTie using default parameters and normalized using FPKM (Pertea et al., 2016). In this study, we normalized and visualized all expression data using TBtools (version 1.098769) software (Chen et al., 2018).

## Results and Discussion

### Identified of *VmLRR-RLKs* in *V. montana*

*V. fordii* and *V. montana* are the two most important main varieties in China. *V. fordii* has high oil content but is susceptible to *Fusarium* wilt, while *V. montana* is resistant to *Fusarium* wilt (Cui et al., 2018). More studies have confirmed that *LRR-RLKs* play important roles in plant stress resistance (Ariza-Suarez et al., 2022; Cao et al., 2021; Geng et al., 2021; Gottin et al., 2021). In the previous study, we only identified and analyzed the *VfLRR-RLKs* in the *V. fordii* due to the lack of the *V. montana* genome (Cao et al., 2021). Identification of *VmLRR-RLKs* from *Fusarium* wilt-resistant *V. montana* and comparison with corresponding members in *Fusarium* wilt-susceptible *V. fordii* will help to further clarify the roles of *LRR-RLKs* in *Fusarium* wilt resistance.

To identify *LRR-RLK* gene family members, we first obtained the Hidden Markov Model to identify *LRR-RLK* members from the Pfam (El-Gebali et al., 2019). At the same time, we also used the LRR-RLK sequence of *V. fordii* as a template and used BlastP software to search the genome of *V. montana*. Subsequently, 243 candidate *VmLRR-RLKs* were found in *V. montana* genome. Three tools, including Pfam (El-Gebali et al., 2019), InterProScan (Jones et al., 2014), and SMART (Letunic & Bork, 2018), were used to determine whether the identified LRR-RLK proteins contained the PK and R domains. Two members were found to lack or not contain the complete LRR-RLK domain. Therefore, this study finally identified 239 *VmLRR-RLKs* in the *V. montana* genome for further analysis (Table S1).

The *LRR-RLK* gene family contains many members, such as 226 members in rice and 236 members in *Arabidopsis* (Hwang, Kim & Jang, 2011). Further studies determined that the number of *LRR-RLK* family members was not necessarily related to genome size. For example, *V. fordii* and *V. montana* have almost the same genome size (Cui et al., 2018; Zhang et al., 2019), but *V. fordii* only had 167 members (Cao et al., 2021), while *V. montana* did contain 239 members. Interestingly, the number of *LRR-RLK* members in cassava and rubber trees was about twice as high as that in *V. montana* (Cao et al., 2021). This may be due to the fact that cassava and rubber trees have additionally experienced recent whole-genome duplication events in addition to those shared by Euphorbiaceae (Cui et al., 2018; Mansfeld et al., 2021). These results suggested that the number of *LRR-RLKs* is closely related to duplication events in addition to whether the species is resistant to disease.

### Gene structure analysis of *VmLRR-RLKs* in *V. montana*

**Figure 1:**
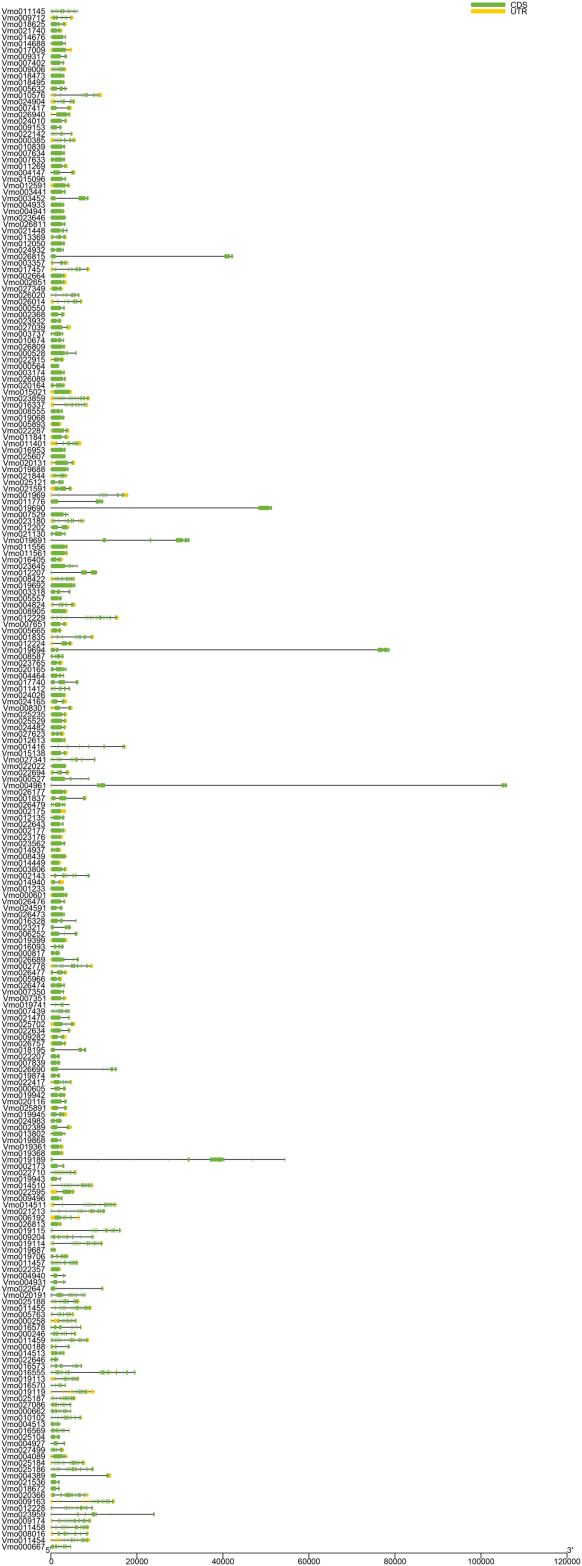
Gene structure of *VmLRR-RLKs* in *V. montana*. The exon-intron organizations of all *VmLRR-RLKs* were visualized by Tbtools software. Introns and exons were plotted by lines and boxes, respectively.

The gene structures are not only closely related to their functions but also may reflect the evolutionary history of gene family members (Cao et al., 2016; Song et al., 2017). In general, ancient genes are generally intronless, and then introns appeared in the long evolutionary process, resulting in more and more complex gene structures (Chen et al., 2020; Liu et al., 2021; Zou, Guo & He, 2011). To gain insight into the evolutionary history of *VmLRR-RLKs* in *V. montana*, we analyzed their exon-intron structures. As shown in Fig. 1, the results showed that the *VmLRR-RLKs* presented a complex gene structure. Four genes, including *Vmo023646, Vmo000564, Vmo025607*, and *Vmo001233*, were intronless genes, suggesting that these genes might be the *VmLRR-RLKs* ancestral genes. *Vmo023859* contained the most introns (28), followed by *Vmo012229* (27) and *Vmo014511* (27), indicating these genes might be young genes. Taken together, our results indicated that each member of *VmLRR-RLK* exhibits a complex genetic structure during the long evolutionary process, which might contribute to the resistance to *Fusarium* wilt in *V. montana*.

### Phylogenetic analysis of *VmLRR-RLKs* in *V. montana*

**Figure 2:**
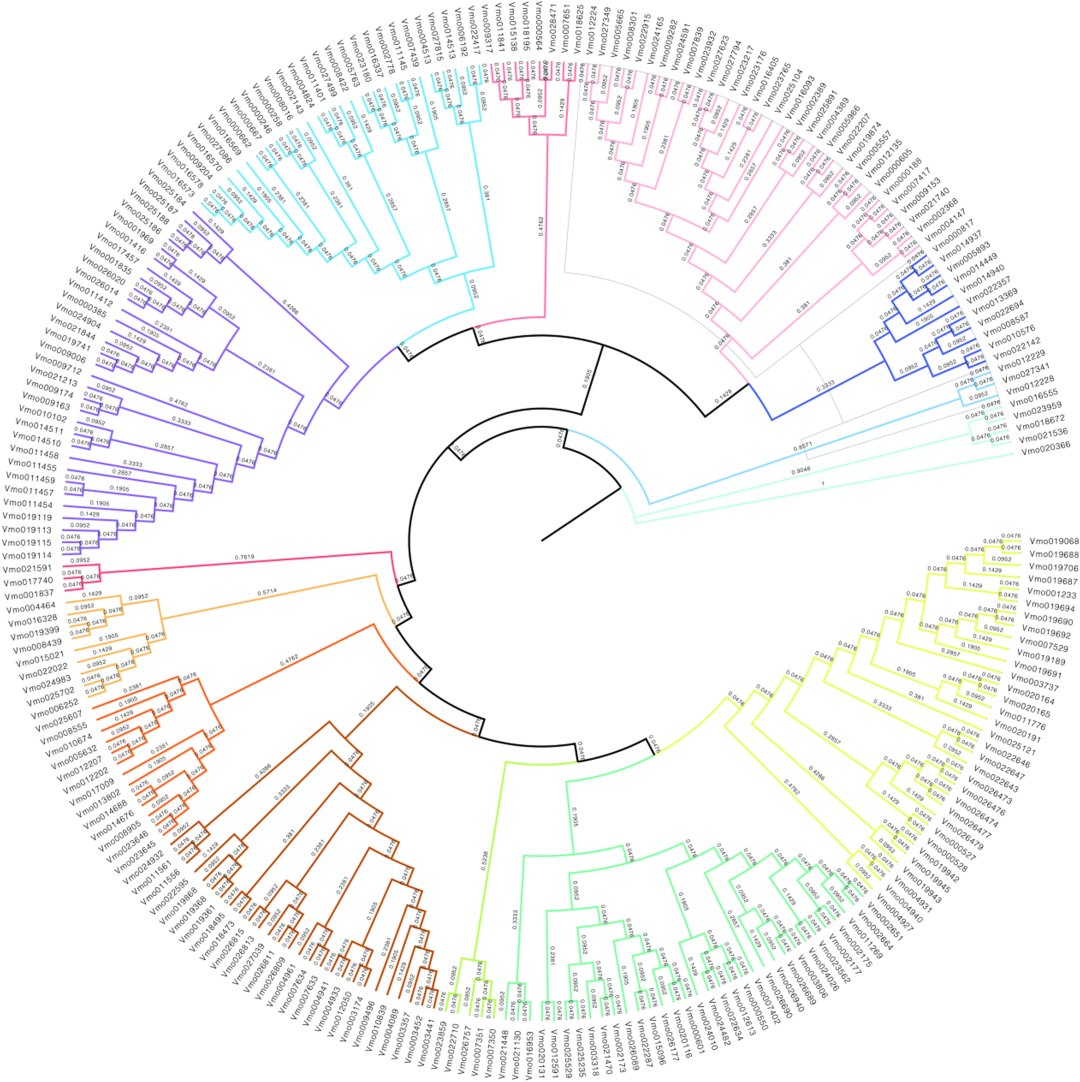
The phylogenetic tree was generated from the alignment result of the full-length amino acid sequences by the neighbor-joining (NJ) method.

In order to further obtain the evolutionary relationships of VfLRR-RLKs in *V. montana*, we first used MAFFT software to conduct multiple sequence alignment analysis of all VmLRR-RLK protein sequences and then used MEGA (version 5) software to construct a phylogenetic tree using the neighbor-joining method. The bootstrap value was used to check whether the constructed evolutionary tree was reliable, as described by Li et al. (2022). As shown in Fig. 2, all *VmLRR-RLKs* were divided into 14 major subgroups, which were named C1 to C14 and distinguished by different colors. This result was basically consistent with the exon-intron distributions, that were, members of the same subgroup contained similar exon-intron structures. Gene duplication and loss events may play important roles in the expansion and contraction of *LRR-RLK* family members (Liu et al., 2016; Magalhães et al., 2016; Zou, Guo & He, 2011). In this study, at the branch tip, the shorter branch length demonstrated the strong amino acid conservation of the cluster genes, suggesting that these members may share the conserved evolutionary relationships, leading them to possibly have similar functions with functional redundancy. In each branch, the two genes from the tip might have undergone a gene duplication event during evolution, or a gene loss event might have occurred.

### The chromosome localization and gene duplication analysis of *VmLRR-RLKs* in *V. montana*

**Figure 3:**
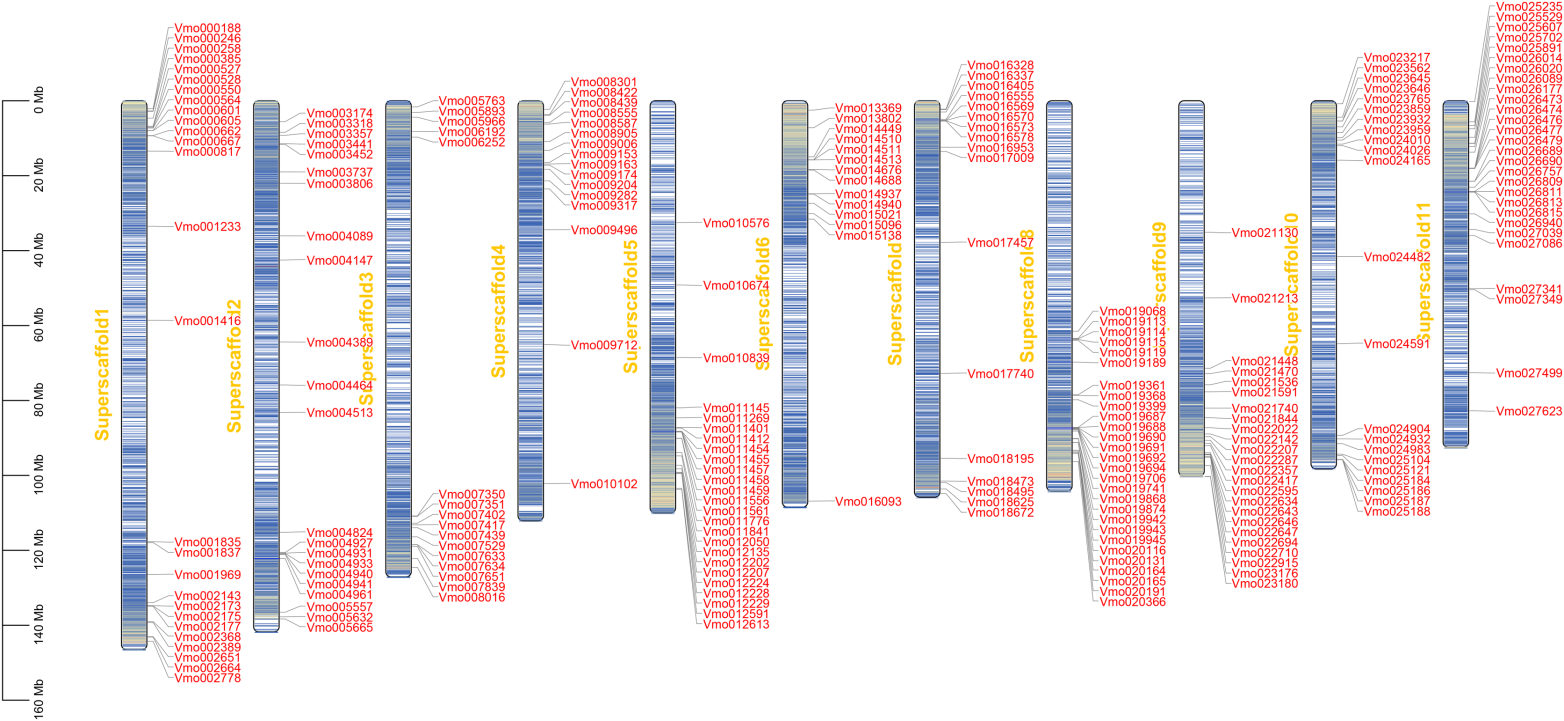
*VmLRR-RLK* genes were mapped to all *V. montana* chromosomes.

Gene location analysis can determine the distribution of genes on chromosomes (Mishra et al., 2021). The chromosome location of each of *VmLRR-RLK* was determined on *V. montana* chromosomes. As shown in Fig. 3, 238 of the 239 *VmLRR-RLKs* were unevenly assigned to chromosomes, while the remaining three *VmLRR-RLKs* were localized on scaffolds in *V. montana* genome. The Vm8 and Vm11 chromosomes each contained 28 *VmLRR-RLKs*, the Vm3 and Vm4 chromosomes each had 16 *VmLRR-RLKs*, the Vm1 chromosome contained 27 *VmLRR-RLKs*, the Vm2 chromosome located 22 *VmLRR-RLKs*, and the Vm5 chromosome contained 25 *VmLRR-RLKs*. There were 14 *VmLRR-RLKs* in Vm6 chromosome, 17 *VmLRR-RLKs* in Vm7 chromosome, 24 *VmLRR-RLKs* in Vm9 chromosome, and 21 *VmLRR-RLKs* in Vm10 chromosome.

**Figure 4:**
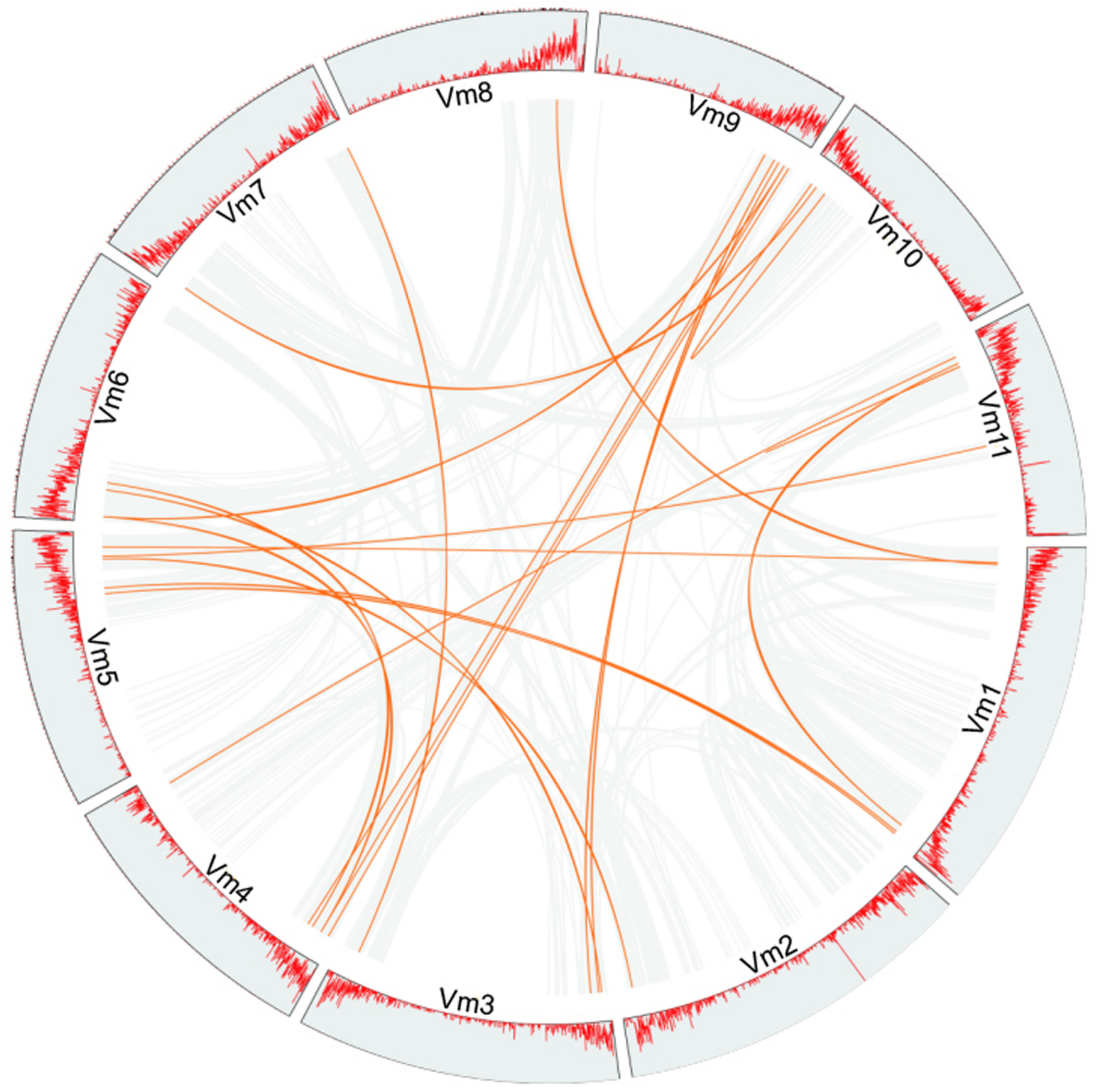
The duplication analysis of *VmLRR-RLKs* in *V. montana*. All putative segmental duplications are linked by the colored lines respectively.

Phylogenetic analysis suggested that gene duplication events might be the main cause of the expansion of members of th*e VmLRR-RLK* gene family in *V. montana*. For example, a total of 15 tandem duplications and 31 segmental duplications were found in cucumber genome (Yu et al., 2022). In *Thinopyrum elongatum* genome, Mishra et al. (2021) identified 191 segmental duplications and 145 tandem duplications, respectively (Mishra et al., 2021). In the potato genome, 16 and 20 genes were predicted to be the results of tandem duplications and segmental duplications, respectively (Li et al., 2018). To further elucidate the expansion mechanism of members of the *VmLRR-RLK* gene family, we analyzed the gene duplication events in *V. montana* (Fig. 4). The results indicated that segmental duplications were the main reason for the expansion of *VmLRR-RLK* gene family members. It is worth noting that this study did not find that members of the *VmLRR-RLK* gene family have undergone tandem duplications in *V. montana*, which was different from the previous results in other plants, such as cucumber, potato, and *T. elongatum* (Li et al., 2018; Mishra et al., 2021; Yu et al., 2022). These data suggested that the evolution mechanisms of *LRR-RLKs* were different in different plant genomes.

### Expression pattern analysis of *LRR-RLK* family genes

**Figure 5:**
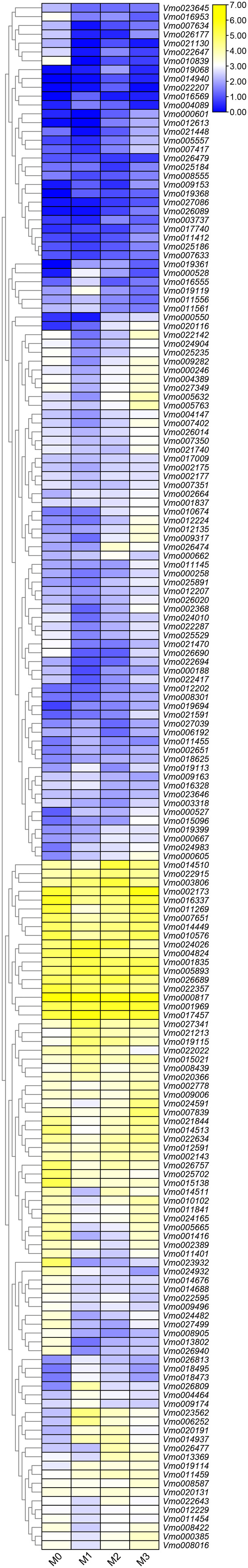
Expression of *VmLRR-RLK* genes during *Fusarium* wilt infection. M0, M1, M2 and M3 indicated the expression of *VmLRR-RLKs* in *V. montana* during the infection stage (0, uninfected stage; 1, 2 days after *Fusarium* wilt infection (dpi); 8 dpi; 3, 13 dpi) by the pathogen *Fusarium* wilt.

More *LRR-RLKs* have been reported involved in disease resistance in plants (Cao et al., 2020; Geng et al., 2021). For example, an LRR receptor-like kinase protein, ERECTA can affect resistance to bacterial wilt controlling development pleiotropically (Godiard et al., 2003). The LRR-RLK/malectin-like IOS1 play important role in BAK1-independent and BAK1-dependent pattern-triggered immunity in *Arabidopsis* (Yeh et al., 2016). XIK1 from *Oryzae pv. oryzae*, an LRR receptor-like kinase gene is involved in Xa21-mediated disease resistance (Hu et al., 2015). In this study, to further determine the functions of the *LRR-RLKs*, we selected the *Fusarium* wilt-resistant *V. montana* and *Fusarium* wilt-susceptible *V. fordii* as the research objects. Among them, the expression data of the *V. fordii VfLRR-RLKs* were derived from a previously published manuscript (Cao et al., 2020, 2021). As shown in Fig. 5, we found that the FPKM values of 26.6% (64/239) *VmLRR-RLKs* genes were <1 response to *Fusarium* wilt infection, proposing that these genes might be expressed in other tissues, such as flowers, roots, and leaves. Remarkably, 22 *VmLRR-RLKs* showed down-regulated expression, while 124 *VmLRR-RLKs* were up-regulated during *Fusarium* wilt infection (Fig. 5).

**Figure 6:**
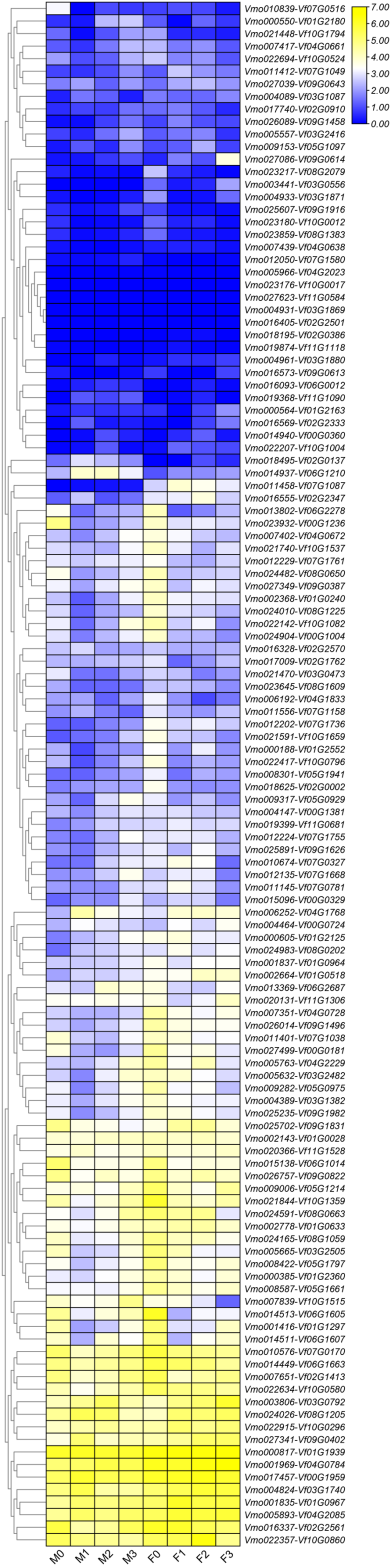
Expression profiles of *LRR-RLK* genes under *Fusarium* wilt infection between *Fusarium* wilt-susceptible *V. fordii* and *Fusarium* wilt-resistant *V. montana*. M0–M3 suggested the expressions of *LRR-RLK* genes in *V. montana* during the infection stage (0, 1, 2, 3) by the pathogen *Fusarium* wilt, and F0–F3 suggested the expression of *LRR-RLK* genes in *V. fordii* during the infection stage (0, 1, 2, 3) by the pathogen *Fusarium* wilt.

Due to the lack of the genome of *V. montana*, in the previous study, we aligned the transcriptome data of *V. montana* infected with *Fusarium* wilt and *V. fordii* infected with *Fusarium* wilt to the genome of *V. fordii* for determine the potential roles of *LRR-RLKs* in resistance to *Fusarium* wilt (Cao et al., 2021). In this study, one-to-one *LRR-RLK* orthologous gene pairs were identified using reciprocal Blast between *V. fordii* and *V. montana* (Cao et al., 2022). Subsequently, we analyzed the expression patterns of these orthologous genes in *Fusarium* wilt-susceptible *V. fordii* and in *Fusarium* wilt-resistant *V. montana* during *Fusarium* wilt infection (Table 1). As shown in Fig. 6, most of the orthologous genes showed opposite expression patterns, of which 55 (44.7%) *LRR-RLKs* pairs were all up-regulated in *V. montana*, while their corresponding orthologous genes were all down-regulated in *V. fordii*; 16 (13%) *LRR-RLKs* pairs were all down-regulated in *V. montana*, while their corresponding orthologous genes were all up-regulated in *V. fordii*. Notably, six orthologous *LRR-RLK* gene pairs (*Vf01G2180-Vmo000550, Vf02G0137-Vmo018495, Vf06G0012-Vmo016093, Vf06G1210-Vmo014937, Vf07G1580-Vmo012050*, and *Vf11G1090-Vmo019368*) were up-regulated in *V. montana*, but were not expressed or expressed relatively low in *V. fordii*, suggesting indicated that these *LRR-RLKs* might play important roles in the resistance of tung tree species to the infection of *Fusarium* spp.

**Table 1:** The one-to-one orthologous relationships of *LRR-RLK* genes between *V. montana* and *V. fordii*.

Gene1	Gene2	Gene1	Gene2
*Vmo000188*	*Vf01G2552*	*Vmo014940*	*Vf00G0360*
*Vmo000385*	*Vf01G2360*	*Vmo015096*	*Vf00G0329*
*Vmo000550*	*Vf01G2180*	*Vmo015138*	*Vf06G1014*
*Vmo000564*	*Vf01G2163*	*Vmo016093*	*Vf06G0012*
*Vmo000605*	*Vf01G2125*	*Vmo016328*	*Vf02G2570*
*Vmo000817*	*Vf01G1939*	*Vmo016337*	*Vf02G2561*
*Vmo001416*	*Vf01G1297*	*Vmo016405*	*Vf02G2501*
*Vmo001835*	*Vf01G0967*	*Vmo016555*	*Vf02G2347*
*Vmo001837*	*Vf01G0964*	*Vmo016569*	*Vf02G2333*
*Vmo001969*	*Vf04G0784*	*Vmo016573*	*Vf09G0613*
*Vmo002143*	*Vf01G0028*	*Vmo017009*	*Vf02G1762*
*Vmo002368*	*Vf01G0240*	*Vmo017457*	*Vf00G1959*
*Vmo002664*	*Vf01G0518*	*Vmo017740*	*Vf02G0910*
*Vmo002778*	*Vf01G0633*	*Vmo018195*	*Vf02G0386*
*Vmo003441*	*Vf03G0556*	*Vmo018495*	*Vf02G0137*
*Vmo003806*	*Vf03G0792*	*Vmo018625*	*Vf02G0002*
*Vmo004089*	*Vf03G1087*	*Vmo019368*	*Vf11G1090*
*Vmo004147*	*Vf00G1381*	*Vmo019399*	*Vf11G0681*
*Vmo004389*	*Vf03G1382*	*Vmo019874*	*Vf11G1118*
*Vmo004464*	*Vf00G0724*	*Vmo020131*	*Vf11G1306*
*Vmo004824*	*Vf03G1740*	*Vmo020366*	*Vf11G1528*
*Vmo004931*	*Vf03G1869*	*Vmo021448*	*Vf10G1794*
*Vmo004933*	*Vf03G1871*	*Vmo021470*	*Vf03G0473*
*Vmo004961*	*Vf03G1880*	*Vmo021591*	*Vf10G1659*
*Vmo005557*	*Vf03G2416*	*Vmo021740*	*Vf10G1537*
*Vmo005632*	*Vf03G2482*	*Vmo021844*	*Vf10G1359*
*Vmo005665*	*Vf03G2505*	*Vmo022142*	*Vf10G1082*
*Vmo005763*	*Vf04G2229*	*Vmo022207*	*Vf10G1004*
*Vmo005893*	*Vf04G2085*	*Vmo022357*	*Vf10G0860*
*Vmo005966*	*Vf04G2023*	*Vmo022417*	*Vf10G0796*
*Vmo006192*	*Vf04G1833*	*Vmo022634*	*Vf10G0580*
*Vmo006252*	*Vf04G1768*	*Vmo022694*	*Vf10G0524*
*Vmo007351*	*Vf04G0728*	*Vmo022915*	*Vf10G0296*
*Vmo007402*	*Vf04G0672*	*Vmo023176*	*Vf10G0017*
*Vmo007417*	*Vf04G0661*	*Vmo023180*	*Vf10G0012*
*Vmo007439*	*Vf04G0638*	*Vmo023217*	*Vf08G2079*
*Vmo007651*	*Vf02G1413*	*Vmo023645*	*Vf08G1609*
*Vmo007839*	*Vf10G1515*	*Vmo023859*	*Vf08G1383*
*Vmo008301*	*Vf05G1941*	*Vmo023932*	*Vf00G1236*
*Vmo008422*	*Vf05G1797*	*Vmo024010*	*Vf08G1225*
*Vmo008587*	*Vf05G1661*	*Vmo024026*	*Vf08G1205*
*Vmo009006*	*Vf05G1214*	*Vmo024165*	*Vf08G1059*
*Vmo009153*	*Vf05G1097*	*Vmo024482*	*Vf08G0650*
*Vmo009282*	*Vf05G0975*	*Vmo024591*	*Vf08G0663*
*Vmo009317*	*Vf05G0929*	*Vmo024904*	*Vf00G1004*
*Vmo010576*	*Vf07G0170*	*Vmo024983*	*Vf08G0202*
*Vmo010674*	*Vf07G0327*	*Vmo025235*	*Vf09G1982*
*Vmo010839*	*Vf07G0516*	*Vmo025607*	*Vf09G1916*
*Vmo011145*	*Vf07G0781*	*Vmo025702*	*Vf09G1831*
*Vmo011401*	*Vf07G1038*	*Vmo025891*	*Vf09G1626*
*Vmo011412*	*Vf07G1049*	*Vmo026014*	*Vf09G1496*
*Vmo011458*	*Vf07G1087*	*Vmo026089*	*Vf09G1458*
*Vmo011556*	*Vf07G1158*	*Vmo026757*	*Vf09G0822*
*Vmo012050*	*Vf07G1580*	*Vmo027039*	*Vf09G0643*
*Vmo012135*	*Vf07G1668*	*Vmo027086*	*Vf09G0614*
*Vmo012202*	*Vf07G1736*	*Vmo027341*	*Vf09G0402*
*Vmo012224*	*Vf07G1755*	*Vmo027349*	*Vf09G0387*
*Vmo012229*	*Vf07G1761*	*Vmo027499*	*Vf00G0181*
*Vmo013369*	*Vf06G2687*	*Vmo027623*	*Vf11G0584*
*Vmo013802*	*Vf06G2278*	*Vmo014513*	*Vf06G1605*
*Vmo014449*	*Vf06G1663*	*Vmo014937*	*Vf06G1210*
*Vmo014511*	*Vf06G1607*		

## Conclusions

The tung tree *LRR-RLK* family members were investigated through gene structure, gene duplication, chromosomal distribution, phylogeny, and expression patterns analysis, which help us to further understand the evolutionary history of this gene family. Our study analyzed the *LRR-RLKs* in *Fusarium* wilt-susceptible *V. fordii* and *Fusarium* wilt-resistant *V. montana* to reveal their expression patterns in response to *Fusarium* wilt infection. Taken together, these data revealed the genetic mechanisms of resistance to *Fusarium* wilt infection and provided important candidate genes for future molecular-assisted breeding in tung tree.

## Supplemental Information

10.7717/peerj.14416/supp-1 Supplemental Information 1 List of *LRR-RLK* genes identified in *V. montana*.

## Additional Information and Declarations

### Data Availability

The following information was supplied regarding data availability: The raw measurements are available in Table 1 and Table S1. The RNA-seq data are available at NCBI: PRJNA483508, PRJNA445068, and PRJNA318350. The genome of tung tree are available at the Genome Sequence Archive at the BIG Data Center, Beijing Institute of Genomics (BIG), Chinese Academy of Sciences: PRJCA000669 and PRJCA001524 that are publicly accessible at http://bigd.big.ac.cn/gsa. These data are also available at NCBI: PRJNA503685 and PRJNA445350.
